# Updates in the treatment of vaginal cancer

**DOI:** 10.1136/ijgc-2021-002517

**Published:** 2022-03-03

**Authors:** Anuja Jhingran

**Affiliations:** Radiation Oncology, The University of Texas M. D. Anderson Cancer Center, Houston, Texas, USA

**Keywords:** vaginal fistula, vagina, vulvar and vaginal cancer

## Abstract

Vaginal cancer is a rare cancer. A lot of the data used in the treatment of this cancer are extrapolated from cervical cancer data. Radiation therapy plays a significant role in the treatment of vaginal cancer. The advances in radiation therapy in both external beam and brachytherapy have improved local control, survival, and toxicity. Brachytherapy plays an important role in treating vaginal cancer, but treatment should be individualized to each tumor. Imaging, particularly magnetic resonance imaging, plays an essential role in the management of patients with vaginal cancer, from diagnosis to staging to treatment management to surveillance.

## INTRODUCTION

Primary vaginal cancer is rare, representing only 10% of all vaginal malignant neoplasms^1^ and only 1–2% of all gynecological cancers.^2^ The definition of primary vaginal cancer excludes any involvement of the cervix and/or vulva as well as any malignant lesion arising in the vagina within 5 years after the treatment of cervical cancer.^3^

Vaginal cancer, like cervical cancer, is strongly associated with the human papillomavirus (HPV).^4^ Risk factors include high-grade squamous intraepithelial lesion as well as smoking and immunosuppression.^5^ It is usually more common in the elderly and postmenopausal women; however, it is rising in younger women due to the increase in persistent HPV infections in regions of high human immunodeficiency virus prevalence.

Squamous cell carcinoma is the most prevalent histology (80%), followed by adenocarcinomas (15%). Melanoma, lymphoma, and sarcoma are rare, comprising the remaining 5%.^6^ Most vaginal cancers arise at the vaginal apex, usually involving the posterior wall.^2^ Lesions in the upper vagina will most likely drain into the pelvic lymph nodes, including the obturator, internal iliac, and external iliac, while lesions in the distal vagina most commonly drain to the inguinal and femoral nodes. Lesions in the mid-vagina may follow both the pelvic and groin node routes.^7^

## IMAGING AND STAGING

Vaginal cancer is staged according to International Federation of Gynecology and Obstetrics (FIGO) staging and is primarily based on clinical findings (Table 1).^8^ Magnetic resonance imaging (MRI) is more sensitive in detecting local tumors, including size, paravaginal, and pelvis wall involvement, because it has superior soft-tissue contrast to computed tomography (CT). Vaginal cancers have intermediate-to-hyper-intense signal intensity on T2-weighted MRI, and the use of water-based vaginal gel can help visualize the tumor better by separating the vaginal walls. Table 1 describes the MRI characteristics per tumor stage and may be helpful when reviewing an MRI.^9 10^ MRI is recommended for diagnosis, local staging, and assessment of recurrence and complication.^9^

**Table 1:** 

	TMN stage			Primary tumor definition	
FIGO stage	Primary tumor	Regional lymph nodes	Distant metastasis	FIGO definition	MRI definition
I	T1	N0	M0	Tumor confined to Vagina, ≤2 cm	Tumor limited to the vaginal wall, shown as an uninterrupted T2-hypointense sub-mucosal layer
II	T2	N0	M0	Tumor invades Paravaginal tissue But not the pelvis wall ≤2 cm	Tumor extends into the paravaginal space or fat, shown as interrupted, hypo-intense vaginal mucosal and muscular layer
III	T3	N0	M0	Tumor extends to Pelvic wall and is any Size and/or hydronephrosis	Tumor invasion of iliac vessels, pelvic muscle, (eg, obturator internus piriformis, and levator ani) or bony structures
III	T1, T2, T3	N1	M0	Tumor extends to Pelvic wall and is any Size and/or Hydronephrosis and spread In the pelvis or groin	
IVA	Any T	Any N	M0	Tumor invades bladder or rectum or Extends beyond pelvis	Tumor invades the adjacent organs involving the mucosal layer of the bladder, rectum, or urethra, or extends beyond the true pelvis
IVB	Any T	Any N	M1		

FIGO, International Federation of Gynecology and Obstetrics; M1, distant metastasis; M, metastasis; M0, no distant metastasis; N0, no regional lymph node metastasis; N1, regional lymph node metastasis; N, lymph node; T, tumor; TNM, tumor, node and metastasis.

As with cervical cancer, positron emission tomography/CT (PET/CT) has been found to be beneficial in detecting nodal metastasis as well as distant metastasis.^11^ One study reported 100% detection of the primary tumor by PET/CT compared with 41% by CT, and 35% detection of enlarged lymph nodes by PET/CT compared with 17% by CT.^11^ It may also be useful in detecting recurrences.

## PROGNOSTIC FACTORS

The most important prognostic factor for vaginal cancer is the stage at the time of diagnosis. In a larger series of patients with vaginal cancer, the 5 year relative survival was 96%, 64–84%, 53–58%, 36%, and 18–36% for stages O, I, II, III, and IV, respectively.^12 13^ Other factors that negatively affect prognosis include tumor size >4 cm, older age, and possibly tumor location outside of the upper third of the vagina.^13 14^ In general, adenocarcinomas have a worse prognosis than squamous cell carcinoma.^13^

Two prognostic factors, high-risk HPV DNA and low MIB-1 index, have been found to have a favorable prognostic value.^15^ MIB-1 index or tumor expression of the proliferation-associated antigen Ki-67 is an immunocytochemical marker of mitotic rate. It has been shown to be important in many gynecological cancers, including vaginal cancers.^15^

## SURGERY

In general, surgery has a limited role in treating vaginal cancer due to the proximity of the cancer to normal tissues such as the bladder, rectum, and urethra. The general recommendation is that surgery might be considered in small stage I tumors (<2 cm in diameter) that are limited to the proximal part of the vagina.^16^

The type of surgery varies depending on where the tumor is and includes local excision, partial vaginectomy, radical hysterectomy, and pelvic exenteration, usually combined with lymph node assessment. In the literature, approximately 25% of patients with stage I-II disease treated with surgery required adjuvant radiation therapy due to positive margin and/or positive lymph nodes.^11 17^ In a series of 124 patients, patients with stage I and II disease had equal survival whether treated with surgery or radiation; however, 55% of the patients received adjuvant radiation therapy after surgery.^18^

Pelvic exenteration may play a role in patients with stage IV disease with recto-vaginal or vesico-vaginal fistula. In this case, the surgery may be done with pelvic node dissection. Another scenario where pelvic exenteration may play a role is when a patient has a central recurrence after radiation therapy.

## RADIATION—EXTERNAL BEAM

Radiation therapy is the treatment of choice in most patients with vaginal cancer, especially in patients with advanced-stage disease. Radiation therapy usually consists of a combination of external beam radiation therapy and brachytherapy. The advantage of radiation therapy is the preservation of the vagina as well as other organs. Brachytherapy alone is not recommended for most tumors, even early-stage, due to a high recurrence rate.^13 18^ External beam is used to treat the primary disease and regional nodes. The purpose is to reduce the volume of the primary vaginal tumor, provide regional lymph node control, and eradicate other microscopic disease.

Over the past two decades, there have been many advances in radiation therapy, both in external beam and brachytherapy, that use and integrate advanced imaging, including CT, MRI, PET/CT, as well as advanced planning to develop more conformal plans that reduce dose to normal tissues while allowing more dose to areas of interest (intensity-modulated radiation therapy or volumetric arc therapy). These new radiation techniques have led to a substantial reduction in dose to normal tissue leading to lower acute and chronic toxicity from radiation therapy.^19–21^

**Figure 1:**
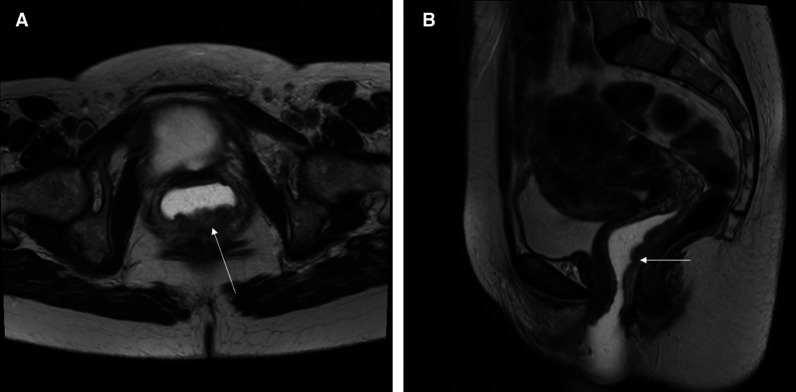
This is a T2 weighted MRI of a patient with vaginal cancer. The lesion shown by the arrow in the posterior wall of the vagina is biopsy-positive vaginal cancer. The lesion involves the entire length of the posterior vaginal wall up to 0.5 cm from the cervix. There is vaginal water base gel in the vagina which shows up as white and separates the vaginal walls so that the lesion can be seen easier.

**Figure 2:**
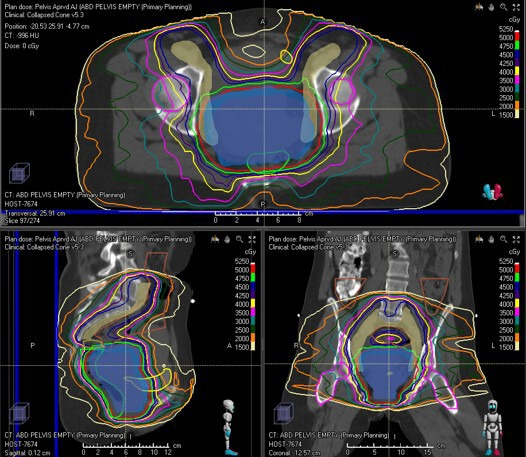
Volumetric arc therapy for the patient with posterior wall vagina cancer seen in Figure 1. The top image is the axilla view showing the nodal clinical volume (CTV) contours in mustard and the vaginal internal gross tumor volume (GTV) in blue. The nodal CTV which includes the inguinal nodes is receiving 45 Gy and the vaginal GTV is receiving 50 Gy. Normal tissues that are outlined include the bladder in yellow and the rectum in green. The bottom right image is the sagittal view of the same, and bottom left is the coronal view of the same plan.

With these highly conformal plans, it is essential to know the full extent of the disease prior to planning and using imaging to help with the planning, so nothing is missed. MRI pretreatment is important for identifying the full extent of the primary disease. Figure 1 shows an MRI of a patient with posterior wall vaginal cancer that extends from about 0.5 cm from the cervix down to the distal 1/3 of the vagina. No nodal involvement was found on the MRI or the PET/CT. Figure 2 shows the volumetric arc therapy used in treating the patient with external beam. The plan includes all nodal regions at risk, including the inguinal nodes as well as the primary tumor. For planning purposes, MRI and PET/CT are often fused to the planning CT to help identify disease, or patients can be simulated on an MRI scanner and planning can be done directly on the MRI.

## CONCURRENT CHEMORADIOTHERAPY

Concurrent chemotherapy and radiation therapy (CCRT) has been adopted in treating vaginal cancer from the data extrapolated in patients with locally advanced cervical cancer. A Cochrane review showed a 6% reduction in the absolute risk of death and an 8% absolute disease-free survival benefit in favor of CCRT in patients with cervical cancer.^22^ A randomized study cannot be undertaken on vaginal cancer due to the rarity of the disease. However, a large US National Cancer Data Base (NCDB) study showed that CCRT was an independent prognostic factor for better overall survival (56 months for CCRT vs 41 months for radiation therapy).^23^ The most common regimen that is used is weekly cisplatin at 40 mg/m^2^; however, other drugs and combinations have also shown benefit.^24–26^

## BRACHYTHERAPY

Brachytherapy is an essential component in the treatment of vaginal cancer. During brachytherapy, the residual disease is treated with a radioactive source (usually Ir192) placed directly or near the disease. This way, the residual disease gets an extra dose while the normal tissues are spared. Two studies from large databases have reported a decrease in survival with the elimination of brachytherapy in the treatment of vaginal cancer. An extensive database study published by the Surveillance Epidemiology and End Results (SEER) found that patients with vaginal cancer treated with brachytherapy had a median overall survival (6.1 years) that was more than 2 years longer than patients who did not receive brachytherapy (3.6 years).^27^ On a multivariant analysis, the survival benefit associated with brachytherapy was independent of FIGO stage, tumor size, and histological type. Patients with tumor size >5 cm had the most significant benefit from brachytherapy.^27^ Another study looking at the NCDB database with patients treated with only CCRT for vaginal cancer also found that brachytherapy boost was associated with an improved 5 year overall survival (62.9% vs 49.3%, p=0.0126).^28^ These findings are similar to results from large database studies in cervical cancer.^29–31^

## TECHNIQUES

**Figure 3:**
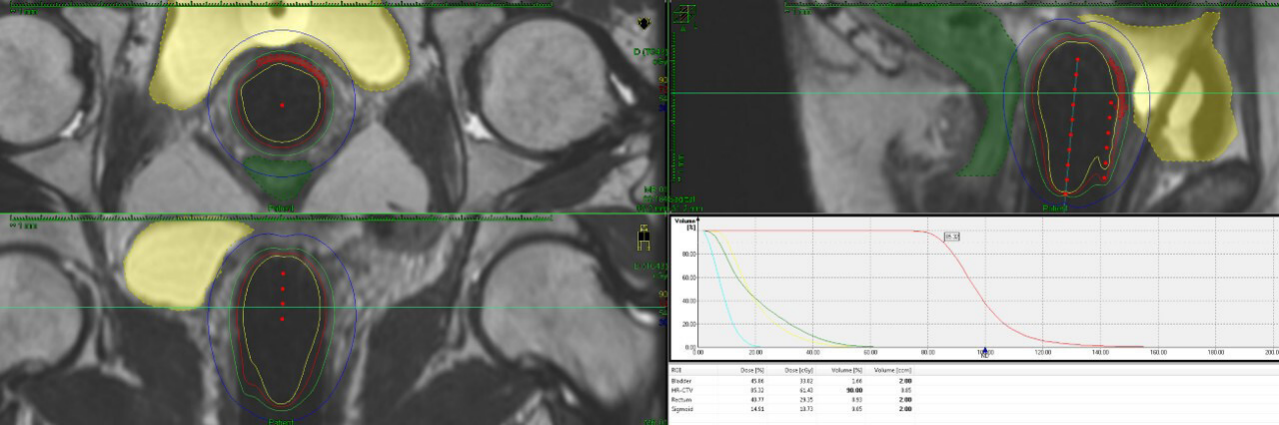
This figure shows an implant for a patient with a left side vaginal lesion that was <5 mm in depth. A multiple channel cylinder was placed and the needles on the left side as well as the central channel were activated. The high-risk clinical treatment volume received 40.54 Gy from the implant giving it a total of 86.54 Gy with a combination of external beam and brachytherapy. The bladder received 21.79 Gy from the implant for a total of 67.79 Gy, the rectum received 19.37 Gy from the implant for a total of 65.37 Gy, and the sigmoid—which was well away from the implant—received 7.08 Gy from the implant to give it a total of 53.08 Gy.

**Figure 4:**
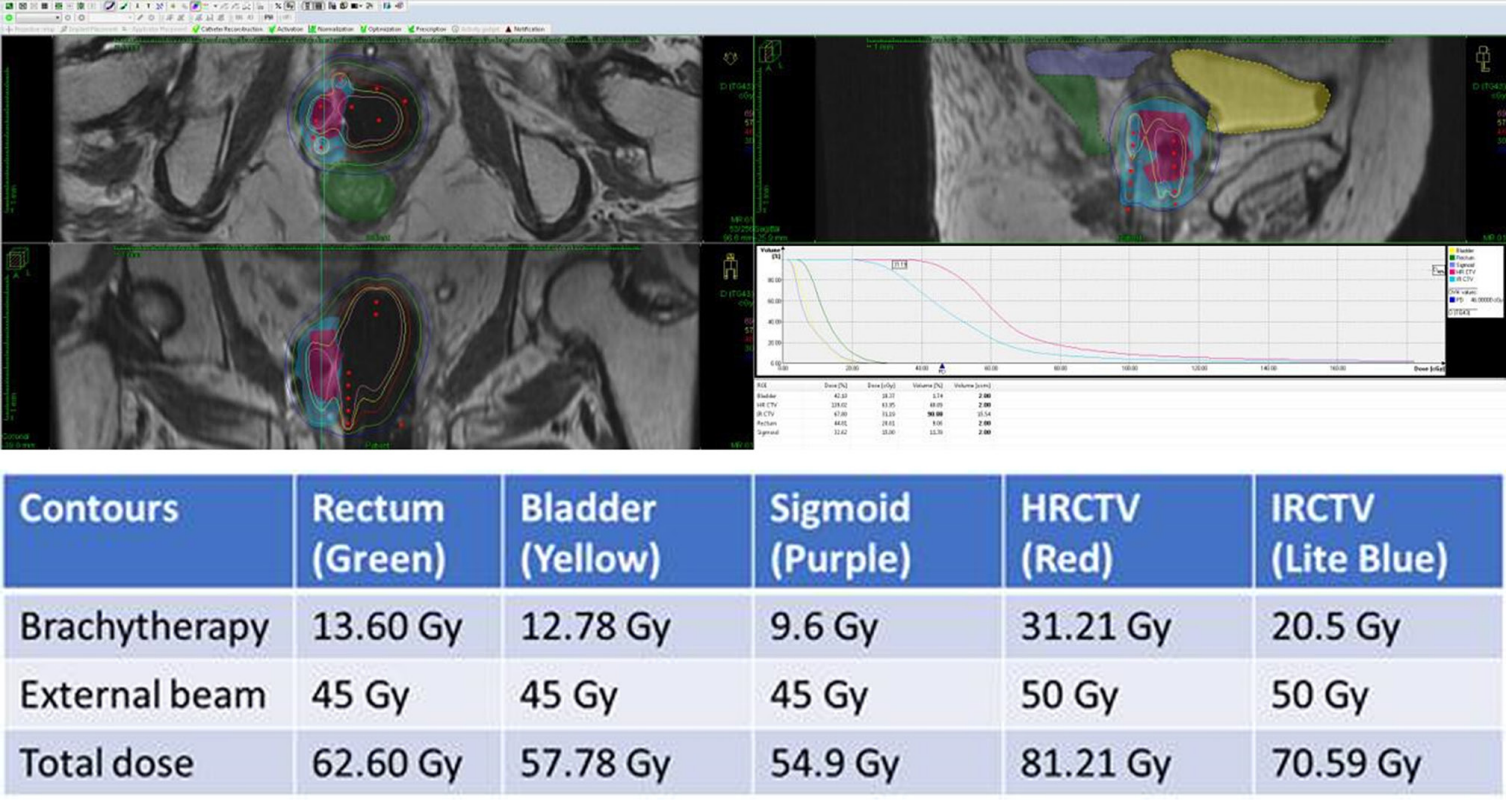
This figure shows an implant of a patient with right side vaginal tumor. The patient still had residual disease after external beam and therefore a multi-channel cylinder as well as free-hand interstitial needs were used. The needles were placed into the right vaginal wall through the perineum using ultrasound guidance. The planning was done using MRI and CT scan.

Brachytherapy techniques vary depending on the tumor’s response and the site of the disease. Intracavitary brachytherapy may be used for superficial tumors (<5–7 mm in diameter). For very superficial lesions, a normal cylinder may be enough; however, for most cases, a multi-channel cylinder will give better coverage and depth dose (Figure 3). For tumors that are more advanced (anything thicker than 7 mm in diameter), a combination applicator must be used. The combination includes a cylinder and needles. The needles can be placed via the perineum either free-hand (Figure 4) or with a perineal template. Adding needles will increase the depth dose without increasing the dose to normal tissues like the bladder and rectum. Several methods can be utilized during the implant to ensure that the applicator and needles are in the correct position, including ultrasound, CT, MRI, or laparoscopic surgery. The combined dose (external beam plus brachytherapy) that is recommended should be above 70 Gy.^14^

## IMAGING AND ADAPTIVE BRACHYTHERAPY

As with external beam, there have been significant advances in brachytherapy over the last couple of decades, and these advancements come in the way of imaging guidance. Historically, brachytherapy was done in two dimensions using orthogonal x-rays and point doses with uniform methods for planning. Over the last two decades, brachytherapy has moved towards image-guided brachytherapy using both or either CT scan or MRI scanning (three-dimensional planning). CT scan helps with verification of the applicator placement and volumetric delineation of organs at risk, but the delineation of the primary tumor remains a challenge due to poor soft-tissue contrast. The dose can be shaped around the probable target volume and organ risks with CT guidance. MRI is the best modality to use during brachytherapy, especially in vaginal cancer where the applicator, primary tumor, and organs at risk are visible, allowing for optimal dose planning. However, even with MRI planning, it is important to know the full extent of disease from the start, including the extent of mucosal spread that sometimes cannot be fully seen by MRI. Placement of fiducial markers at the start of the external beam to mark the borders of the tumor can be helpful when planning brachytherapy. It may also be beneficial during external beam planning.

Three-dimensional image-guided brachytherapy has led to a 10% absolute gain in survival compared with two-dimensional brachytherapy in patients with cervical cancer.^32^ Toxicity has also decreased using imaged guided brachytherapy compared with two-dimensional brachytherapy.^32 33^ Similar to cervical cancer, GEC-ESTRO (Groupe Européen de Curiethérapie (GEC) and the European SocieTy for Radiotherapy & Oncology (ESTRO)) has developed terminology and doses for vaginal cancer. These recommendations were published in 2019.^34^ Several small studies using GEC-ESTRO concepts have published their outcome data for patients with vaginal cancer and have shown an improvement in local control, survival, and toxicity, though the follow-up is much shorter using three-dimensional image-guided adaptive brachytherapy.^24 35–39^ In general, with a median clinical target dose of 79 Gy (range 73–86 Gy), the local control ranges from 82–93% and 2 year overall survival from 62–91%. The severe toxicity was also less than two-dimensional, ranging from 2–23%.^10^

## EXTERNAL BEAM BOOST

For some tumors that may be too large or do not have favorable anatomy for brachytherapy, external beam may be used as a boost. Doses of 66 to 70 Gy can be delivered safely using conformal fields without much toxicity. In an extensive series of patients with vaginal cancer, Frank et al reported 76% local control and 67% disease-specific survival in 5 years in 63 patients (32%) with vaginal cancer treated with external beam alone doses of 66–70 Gy.^13^ The authors concluded that though brachytherapy plays an important role in the treatment of many patients with vaginal cancer, it should be selected carefully, and treatment should be individualized depending on the site and size of the tumor at presentation and response to treatment.^13^ With new technology on the horizon, including linear accelerators that use MRI for daily imaging and alignment, the use of external beam boost in patients may be an even more viable option for patients who cannot receive brachytherapy in the future. With MRI, more accurate alignment and smaller margins can be used. Therefore, dose may be escalated without increasing the dose to the normal tissues, including the rectum and bladder. However, long-term data are needed before these technologies become standard in treating vaginal cancer.

## LOCAL RECURRENCE

Local recurrences in vaginal cancer are common, occurring in 23–26% of patients at 5 years, and about 80% of them occur within the first 2 years and 90% at 5 years.^10 40^ The primary predictor for local recurrence is the stage. The reported recurrence rate is 24% for stage I, 31–32% for stage II, 53% for stage III, and 73–83% for stage IV.^40^ There is conflicting evidence on other risk factors such as lesion location, grade, and HPV status. The prognosis of patients who have a recurrence is poor, with survival correlated with stage. Patients with stage I/II with a recurrence have a 12–18% survival rate compared with 0–3% survival rate for initially stage III/IV disease patients.^40^

Patients with local recurrence do better than patients with distant metastasis (20% vs 4% at 5 years).^40^ MRI is a useful tool to use to follow patients for recurrence versus complications. T2 weighted imaging on MRI at 12–18 months after treatment can differentiate between tumor recurrence and fibrosis, with the tumor being hyperintense on T2.^41^ PET/CT may be useful in assessing for recurrent disease, but MRI is a more helpful tool in determining tumor infiltration and volume.

## COMPLICATIONS

It is challenging to evaluate complications, especially in series that used two-dimensional treatment planning for brachytherapy and external beam. The general rate of severe late toxicity (grade 3 or higher) ranged from 8% to 30%, with severe gastrointestinal and genitourinary morbidity being the most frequent. In the large series by Frank et al, the 5 year and 10 year cumulative rate of major complications rate was 10% and 17%, respectively.^13^ The majority of the complications were gastrointestinal (76% of all complications), and these included proctitis requiring transfusion (seven patients), fistula (five patients), small-bowel obstruction (four patients), large-bowel obstruction (one patient), rectal ulcer (one patient), and incontinence (one patient). Eight of the 11 rectal toxicities occurred in patients with posterior wall disease. The genitourinary complications included one urethral complication and five bladder complications, including two vesicovaginal fistulae and three cases of hemorrhagic cystitis. All five cases of bladder complications happened in patients with anterior wall disease.^13^

There appear to be fewer reports of severe toxicity for patients treated with adaptive image-guided brachytherapy than with two-dimensional brachytherapy. Most patients treated with image-guided brachytherapy were also treated with intensity-modulated radiation therapy/volumetric arc therapy. The studies report 2–23% severe late toxicity with a median 3-year follow-up.^10^

Imaging is important in documenting complications. MRI is probably the best imaging tool available in evaluating causes of symptoms in patients, most likely due to complications. MRI is especially helpful in evaluating for fistula with a 91% accuracy rate, which is best seen in T2 weighted images.^42^ MRI is also beneficial, as discussed earlier, in determining whether vaginal scarring is due to vaginal fibrosis or recurrence. Imaging in general—whether it is MRI or CT scan—is excellent in documenting cystitis, proctitis, bowel strictures and perforation, pelvic bone osteonecrosis, and stress fractures, and should be used for evaluation of patients with symptoms.

## CONSIDERATIONS AND FUTURE DIRECTIONS

Vaginal cancers are rare, but most patients are treated with CCRT. As radiation therapy advances with image guidance in both external beam and brachytherapy, the outcome in both local control and overall survival is improving, and toxicities are decreasing, but studies are small with short follow-up. More extensive studies with longer follow-up are needed. To this end, an international study on primary chemoradiation using image-guided external beam and brachytherapy to treat vaginal cancer is being initiated using common terminology.

Brachytherapy has proven to be a prognostic factor in the treatment of vaginal cancer. A recent study done using the NCDB showed a significant decline in the use of brachytherapy from 2004 to 2011 (19.1%), which was mirrored by increased utilization of intensity-modulated radiation therapy in the same time period. The use of brachytherapy was significantly associated with facility volume, younger age and lower stage, and academic institution versus community practice.^43^ Another study done through the SEER database found a decrease in the use of brachytherapy in patients with vaginal cancer by the rate of 0.5% per year.^27^ The factors associated with the omission of brachytherapy in this study included age, histology that was not squamous cell type, tumors larger than 5 cm in diameter, and advanced-stage disease (anything beyond stage I).^27^ This trend is similar to the trend observed in the treatment of locally advanced cervical cancer. The decrease in the use of brachytherapy for these cancers is very problematic, especially in the face of the survival advantage of brachytherapy over the most conformal external beam techniques. Heightened awareness of this trend as well as its causes—including a decrease in patient volume of these cancers due to HPV vaccines and screening, the complexity of these procedures, and inadequate training of residents—is needed to prevent a further decline in the use.^44^

Economic factors and the complexity of the procedures, including personnel cost and machine cost, have imposed barriers to the development, uptake, and evolution of brachytherapy in the treatment of gynecological cancers. However, data show that scaling up the use of both external beam and brachytherapy for cervical cancer over the next 20 years could save almost 12 million life-years. In addition, an economic benefit of nearly US$60 billion is predicted, despite the growth of HPV vaccination programs over the time period.^45^ Image-guided brachytherapy is considered to be cost-prohibiting due to high personnel and infrastructure costs in many parts of the world compared with two-dimensional brachytherapy. However, in a Canadian analysis, MRI-guided brachytherapy improved clinical outcomes. It saved more money than two-dimensional brachytherapy from the perspective of the public health payer by avoiding the downstream costs of managing cancer recurrence and treating side effects.^46^ The use of three-dimensional printing to develop personalized devices may also help reduce the cost of equipment needed to treat patients and help increase the precision of brachytherapy treatment.^47^

Apart from the improvement in radiation therapy, further improvement in systemic therapy is needed, and these data likely need to be extrapolated from cervical cancer data. These data include the use of immunotherapy and other targeted agents that are being developed. The most significant difference that can be made in decreasing the incidence of vaginal cancer by prevention comes from improved dissemination of HPV vaccines throughout the world.
